# Electrophysiological Profiling of Neocortical Neural Subtypes: A Semi-Supervised Method Applied to *in vivo* Whole-Cell Patch-Clamp Data

**DOI:** 10.3389/fnins.2018.00823

**Published:** 2018-11-13

**Authors:** Parviz Ghaderi, Hamid Reza Marateb, Mir-Shahram Safari

**Affiliations:** ^1^Neuroscience Research Center, Shahid Beheshti University of Medical Science, Tehran, Iran; ^2^Biomedical Engineering Department, Engineering Faculty, University of Isfahan, Isfahan, Iran; ^3^Brain Science Institute, RIKEN, Wako, Japan; ^4^Brain Future Institute, Tehran, Iran

**Keywords:** *in vivo* whole-cell patch-clamp, cell-type classification, discrete cosine transform, parvalbumin, somatostatin, pyramidal, vasoactive intestinal peptide, 5HT3a

## Abstract

A lot of efforts have been made to understand the structure and function of neocortical circuits. In fact, a promising way to understand the functions of cortical circuits is the classification of the neural types, based on their different properties. Recent studies focused on applying modern computational methods to classify neurons based on molecular, morphological, physiological, or mixed of these criteria. Although there are studies in the literature on *in vitro*/vivo extracellular or *in vitro* intracellular recordings, a study on the classification of neuronal types using *in vivo* whole-cell patch-clamp recordings is still lacking. We thus proposed a novel semi-supervised classification method based on waveform shape of neurons' spikes using *in vivo* whole-cell patch-clamp recordings. We, first, detected spike candidates. Then discriminative features were extracted from the time samples of the spikes using discrete cosine transform. We then extracted the center of clusters using fuzzy c-mean clustering and finally, the neurons were classified using the minimum distance classifier. We distinguished three types of neurons: excitatory pyramidal cells (Pyr) and two types of inhibitory neurons: GABAergic- parvalbumin positive (PV), and somatostatin positive (SST) non-pyramidal cells in layer II/III of the mice primary visual cortex. We used 10-fold cross validation in our study. The classification accuracy for PV, Pyr, and SST was 91.59 ± 1.69, 97.47 ± 0.67, and 89.06 ± 1.99, respectively. Overall, the algorithm correctly classified 92.67 ± 0.54% of the cells, confirming the relative robustness of the discriminant functions. The performance of the method was further assessed on *in vitro* recordings by using a pool of 50 neurons from Allen institute Cell Types Database (5 major subtypes of neurons: Pyr, PV, SST, 5HT3a, and vasoactive intestinal peptide (VIP) cells). Its overall accuracy was 84.13 ± 0.81% on this data set using cross validation framework. The proposed algorithm is thus a promising new tool in recognizing cell's type with high accuracy in laboratories using *in vivo*/*vitro* whole-cell patch-clamp recording technique. The developed programs and the entire dataset are available online to interested readers.

## Introduction

Neocortex is one of the most complex structures of the brain, mainly involved in higher brain functions. It is composed of many functionally differentiated components. A lot of efforts have been made to understand the structure and function of neocortical circuits, in the literature (Markram et al., 2004; Tremblay et al., 2016). Classifying neocortical neural types is a critical stage in creating a neocortical circuit model (Santana et al., 2013). Neuronal type classification is a hot topic in neuroscience with many open questions. Some of which were considered in the European Human Brain Project and the American BRAIN initiatives as the first step of the project (Markram, 2012; Insel et al., 2013). Several types of neurons were classified based on three independent features: physiological__ the firing patterns of the cells, morphological__ the anatomical structure of dendrites and axons, or molecular__ the profile of gene expression (Armañanzas and Ascoli, 2015). Recent studies focused on applying modern computational methods to classify neurons based on molecular (Sugino et al., 2006; Schulz et al., 2007; Cahoy et al., 2008), morphological (Guerra et al., 2011; Sümbül et al., 2014), physiological (Li et al., 2012; Battaglia et al., 2013; Druckmann et al., 2013), or their combinations (Karagiannis et al., 2009; McGarry et al., 2010). The cerebral cortex is mainly composed of two types of neurons: (1) excitatory pyramidal neurons (~80% of neocortical neurons) and inhibitory non-pyramidal interneurons (~20% of neocortical neurons, Ghani and Yuste, 2014). The pyramidal neurons releasing glutamate as a neurotransmitter, form the main class of excitatory projecting neurons (Baughman and Gilbert, 1981). On the other side, non-pyramidal interneurons, are inhibitory releasing gamma-aminobutyric acid (GABA) as a neurotransmitter. They have a large diversity of somatic, dendritic and axonal morphologies (Tremblay et al., 2016). GABAergic inhibitory interneurons are classified based on the expression of biochemical markers including three calcium-binding proteins, parvalbumin (PV), calbindin (CB), and calretinin (CR). They are also classified based on four neuropeptides, neuropeptide cholecystokinin (CCK), somatostatin (SST), vasoactive intestinal peptide (VIP) and neuropeptide Y (NPY, Cauli et al., 2000). GABAergic inhibitory interneurons (IN) play a crucial role in processing information flow in the cortex. GABAergic INs have critical roles in many cortical functions, such as controlling the excitatory and inhibitory balance in the network and gain control (Rudy et al., 2011; Tremblay et al., 2016). Among all subtypes of GABAergic INs, PV, SST, and 5HT3a (5HT3aR) constitute nearly the entire neocortical interneurons. The PV group, constituting 40% of GABAergic interneurons, includes fast spiking basket and chandelier cells. The SST group, including the Martinotti cells, represents 30% of this population, while the 5HT3aR group, accounts for 30% of this population (Rudy et al., 2011).

The identification accuracy of specific neuronal types based on the spiking features decreases in extracellular recordings. Noise from different sources such as instrumentation noise, and interferences, hinder its accuracy (Becchetti et al., 2012). The patch-clamp technique used for electrophysiological recordings was first developed to record single-channel electrical signals of neurons membrane (Neher and Sakmann, 1976) and was later used to record the macroscopic electrical activity of a single neuron in the so called whole-cell configuration (Hamill et al., 1981). *In vivo* whole-cell patch-clamp recording requires a sequence of delicate expertise by the experimentalist. The main challenges of this technique are stability (i.e., reducing brain movements as well as environmental oscillations), recording duration, and also maintaining the high quality of recordings (Schramm et al., 2014). Thus, proposing a computational method for the classification of different neuronal subtypes using *in vivo* recording is invaluable.

Although a number of studies were proposed in the literature on neuronal classification using electrophysiological recording techniques such as multi-array extracellular recording (Yang et al., 2013), *in vitro* whole-cell patch-clamp (Druckmann et al., 2013; Sümbül et al., 2014) or *in vivo* tetrode recordings (Li et al., 2012), no study was performed to classify neuronal types using *in vivo* whole-cell patch-clamp recordings to the best of our knowledge. In fact, there are some studies focusing on electrophysiological parameters of specific neurons using *in vivo* whole-cell recordings (Descalzo et al., 2005; Azouz and Gray, 2008), but no classifying was performed in such studies.

Therefore, the aim of our study is to design a novel method for identifying neuronal types based on the waveform shape of neurons spike using *in vivo* whole-cell patch-clamp recordings. This approach would be particularly useful for laboratories with intracellular recordings *in vivo* in which 2-photon imaging is not possible. This method might also be beneficial for identifying neuron's subtype from deeper structures, where visualization is challenging and alternative approaches such as blind intracellular recording is an appropriate option. We proposed a method to extract most informative features from spikes. In this method, we calculated Discrete Cosine Transform (DCT) from each spike as representative features. In our study, we distinguish three types of neurons: excitatory pyramidal cells and two types of inhibitory neurons: GABAergic- parvalbumin positive (PV+) and somatostatin positive (SST+) non-pyramidal cells in layers I and II/III of the mice primary visual cortex. The rest of the paper is organized as the following. In the next section, information about the study population, experimental protocol, and our data mining methods is presented. The results of the proposed method are provided in section Results. The discussion is provided in section Discussion, and finally, the conclusions are summarized in section Conclusion.

## Materials and methods

### Dataset

#### Mice preparation

The Animal Experiment Committee of RIKEN BSI (Brain Science Institute; http://www.brain.riken.jp/en/) approved the entire experimental procedures and the experiments were accomplished according to the guidelines of the Committee. The experiments were performed on urethane anesthetized mice. The data and experimental protocol presented in this paper are the same as our recent publication (Safari et al., 2017). It is briefly summarized here. The entire data was recorded from three types of transgenic mouse lines in which PV, SST or whole GABAergic interneurons express Channelrhodopsin-2(ChR2). The following three lines of transgenic mice used in this study:

1. VGAT-ChR2-YFP mice, B6. Cg-Tg(Slc32a1-COP4^*^H134R/EYFP)8Gfng/J).
2. SST-ChR2-YFPmice, STOCK SST < Tm2.1(cre)Zjh > J × B6;129S-Gt(ROSA)26Sortm32 (CAG-COP4^*^H134R/EYFP) Hze/J.
3. PV-ChR2-YFP mice, B6;129P-Pvalbtml (cre)Arbr/J × B6;129S-Gt(ROSA)26Sortm32(CAG COP4^*^H134R/EYFP) Hze/J.

Urethane (1.5–1.9 mg/g body weight), with additional doses if necessary was used to anesthetize the animals. A small custom-made head chamber attached over the occipital region of the left hemisphere, was used to immobilize the animal's head under a two-photon microscope. After removing a small part of the skull and dura mater over the primary visual cortex, the exposed cortex was covered with agarose (1.5–2.0% in Ringer's solution).

#### Electrophysiological recording

**Figure 1:**
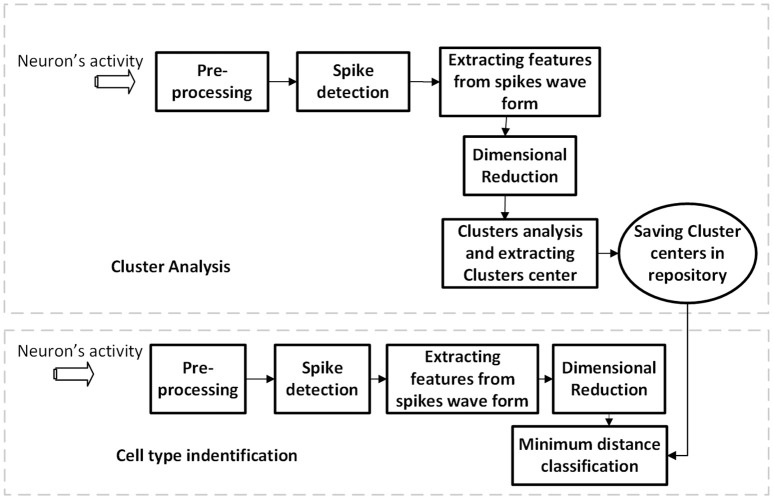
The block diagram of our proposed method for different cell-type identification.

The membrane potentials were recorded using double whole-cell current-clamp simultaneously from YFP-positive interneurons (PV or SST-positive interneurons) and nearby pyramidal (YFP-negative neurons with large soma of pyramidal shape and large apical dendrites) neurons. They were recorded at a depth of 110–230 μm of the cortical surface. The average distances between the PV–SST was 28 ± 5.0 μm while it was 36 ± 6.0 μm for PV- Pyr cell pairs. The multi-clamp amplifier (Multiclamp 700B) was used for recording, and the data was sampled at 20 kHz. Recordings were digitized using NI-DAQ board (PCI-MIO-16E-4, National Instruments) and acquired using custom-made LabVIEW software. The block diagram of our proposed method is shown in Figure 1. It consists of two procedures namely as the cluster analysis and the classification of cell types. The details of the algorithms are discussed as the following.

### Pre-processing and denoising

The third-order digital Butterworth Notch filter with the cut-off frequency of 50 Hz with 5 Hz of bandwidth in the forward and reverse directions was used to remove power line interference from the signals. The quality of the recorded signals was further improved using a moving average filter (the window length of 0.4 ms).

### Extracting features and classification

The proposed features were extracted from the time samples of the spikes. This procedure was performed in several steps illustrated as below:

#### Single-spike extraction

**Figure 2:**
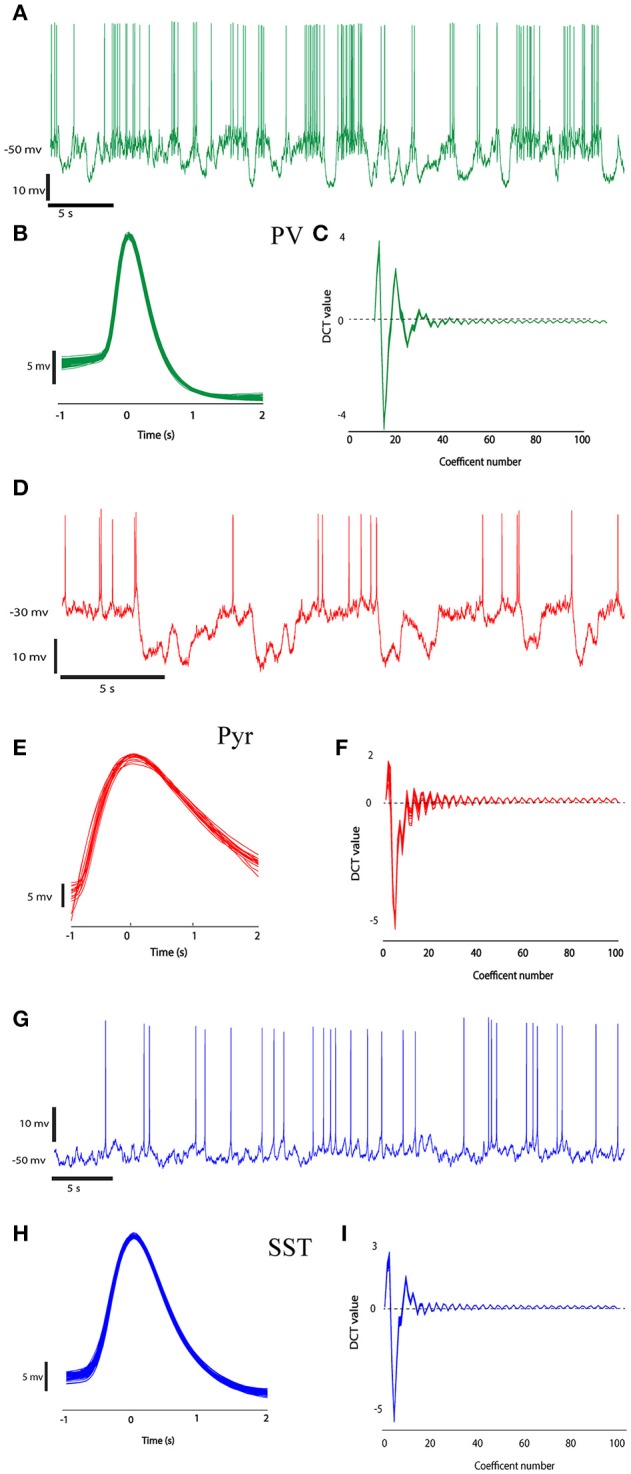
The recorded data from three different neuron types (PV, Pyr, and SST). Recorded raw data **(A,D,G)** and the ensemble of spikes extracted from the raw data by windowing over the spikes' peaks at second column **(B,E,H**, 1 ms before peak to 2 ms after each spike peak). **(C,F,I)** shows DCT coefficients extracted from their spikes waveform.

First, single spikes were extracted from the signal using segmentation. This was performed by thresholding the trace of neurons activity. Times of the peak of each action potential were determined using a simple threshold procedure. The time of the peak occurrence of each spike was used as a marker. The spikes were extracted using temporal windows with the length of 3 ms around the markers (−1 to +2 ms around peaks location; Perrenoud et al., 2016). Figure 2 shows sample traces of different types of neurons with the pool of extracted spikes. With a visual judgment, variability of spike waveforms of different types of neurons is apparent.

#### Discrete cosine transform

Each epoch in our study had 60 samples (i.e., 3 ms epoch width multiplied by the sampling rate of 20 kHz). Such epochs were zero-padded to 100 samples. The DCT coefficients of each detected spike were used as the feature vector. The DCT, first introduced by Ahmed et al. (1974), has been used in the literature for feature extraction (Rao and Yip, 1990; Dabbaghchian et al., 2010). For an input time series *u(n)* (i.e., the time samples of each detected spike) with the length *N* (i.e, 100 in our study), its DCT, *v(k)*, is calculated as below (Hafed and Levine, 2001):

(1) $$v(k)=\alpha (k)\sum_{n=0}^{N-1}u(n)\cos\left(\frac{(2n+1)\pi k}{2N}\right)\;0\leq k\leq N-1$$

where

(2) $$\alpha (k)=\sqrt{\frac{k+1}{N}};0\leq k\leq N-1$$

Thus, 100 DCT coefficients i.e., *v(0)* …,*v(99)* were extracted for each segment (i.e., spike) and used as features for the rest of the analysis. Such a number of DCT coefficients were used based on the preliminary analysis as to have the best class separation in our study.

#### Action potential shape features

**Figure 3:**
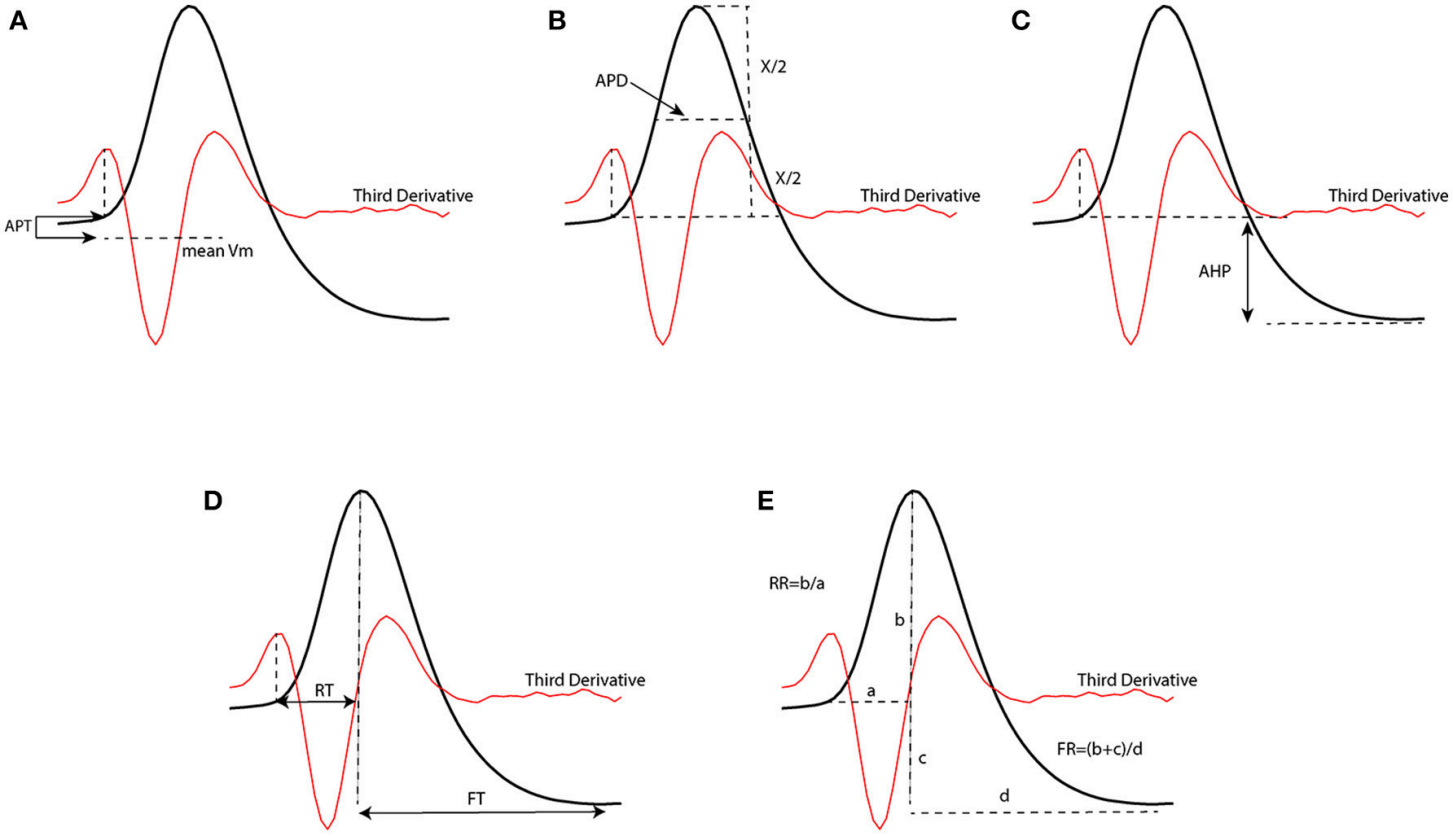
Extracted parameters from an action potential waveform which have been used to classify neurons. [APT, action potential threshold **(A)**; APD, action potential duration **(B)**; AHP, After hyperpolarization **(C)**; RT, rise time **(D)**; FT, fall time **(D)**; RR, rise rate **(E)**; FR, fall rate **(E)**].

In order to test the performance of our proposed method to classify neural subtypes with previously proposed method in literature (McGarry et al., 2010; Zaitsev et al., 2012; Helm et al., 2013), we calculated some features related to the shape of action potentials. We thus extracted 7 electrophysiological variables from the waveform of the action potential. Such extracted features were: action potential threshold (APT), action potential duration (APD), after hyperpolarization (AHP), rise time (RT), fall time (FT), rise rate (RT), and fall rate (FR). Extracted parameters from a sample action potential are depicted in Figure 3.

### Principal component analysis

Working on high dimensional data is challenging in machine learning. In fact, different problems happen when the features have high dimensions (Theodoridis and Koutroumbas, 2009). One of the well-known methods for dimensionality reduction is the Principal Component Analysis (PCA). The PCA is well-described and reviewed in the literature (Bishop, 2006). The PCs are formed by decomposing the correlation matrix of data to eigenvalue. The eigenvectors (i.e., uncorrelated vectors) corresponding to each eigenvalue is called principal components (PCs). The dimension of the dataset is usually reduced using PCA since the number of uncorrelated components is always less than that of the original variables. The extracted spike features were represented as a matrix of feature, in which each row and column is related to a spike and DCT coefficients, respectively. We performed PCA on 100 DCT coefficients and kept two PCs for further analysis corresponding with the cumulative percentage variance (CPV) of 90%.

### Cluster analysis

In this step, we used 90 percent of the date (feature's matrix) for clustering and saved the center of each cluster in the repository as depicted in Figure 1. We used the fuzzy c-mean (FCM) clustering method to determine clusters and their centers (i.e., cluster representatives). The FCM algorithm is one of the most popular clustering algorithms developed by Dunn in 1973 (Dunn, 1973). In this clustering method, each sample of data belongs to many clusters with different membership value. In fact, in this step, we extracted all centers of clusters using 90% of data and the remaining 10% was used to validate our model.

### Cell type identification

In the cell type identification phase, we used the remaining percentage (10 %) of the data to classify each sample (spike) to different types using the minimum distance classifier (Duda et al., 2000). A given pattern *X* = (*x*_1_, *x*_2_, …, *x*_*N*_) of unknown classes is classified into the class no. *i* if its Euclidean distance (D_i_ in equation 3) to the center *i* [*C*_*i*_ = (*c*_*i*, 1_, *c*_*i*, 2_, …, *c*_*i, N*_)] is smaller than those to all other centers.

(3) $$D_{i}=\left\|X-C_{i}\right\|=\sqrt{\sum_{j=1}^{N}{\left(x_{j}-c_{i,j}\right)}^{2}}$$

where D_i_ is the Euclidean distance between X and the ith cluster's Center, x_j_ is the value of X in the jth dimension, and c_i, j_ is value of C_i_ in the jth dimension.

### Class separability based on scatter matrices

To identify whether DCT transformation improved the class separability, J_3_ index was used in our study (Theodoridis and Koutroumbas, 2009). Class scatter matrices for class separability is defined as:

(4) $$\begin{array}{l} S_{b}=\sum_{i=1}^{c}{\left(m_{i}-m_{j}\right)}^{t}(m_{i}-m)\\ S_{w}=\sum_{i=1}^{c}{\sum^{\text{​}}}_{j=1}^{ni}{\left(x_{ij}-m_{j}\right)}^{t}(x_{ij}-m_{i}) \end{array}$$

where, C is the number of classes, ni is the number of samples in the class *i*, m_i_ is the mean of instances in the class *i* and m is the average of all samples. X_ij_ is the sample *j* in the class *i*, S_b_ is the between class scatter matrix and S_w_ is the within class scatter matrix (where Sb>>Sw). The J_3_ index is then defined as below:

(5) $$\begin{array}{r} J_{3}=trace\left(S_{b}+S_{w}\right)/trace\left(S_{w}\right) \end{array}$$

where, the trace is the sum of the eigenvalues. The criterion J_3_ is an unbounded measure. The larger the value of J_3_, the smaller the within class scatter as compared to the between class scatter and thus shows better class separability.

### Classification performance evaluation

The 10-fold cross-validation framework was used to assess the performance of the proposed classification method. The feature matrix was divided into 10 equal size parts. In each fold, the cluster centers were determined by 90% of the data in the training phase (determining clusters center), while the remaining 10% was used to classify using the minimum distance classification (Duda et al., 2000). This process was repeated for the entire folds. The spike identification performance was assessed for each class in terms of precision, recall and accuracy as the following:

(6) $$precision_{i}(PR_{i})=\frac{TP_{i}}{TP_{i}+FP_{i}}$$

(7) $$Recall(RL_{i})=\frac{TP_{i}}{TP_{i}+FN_{i}}$$

(8) $$\begin{array}{r} Accuracy_{i}\left(Acc_{i}\right)=\frac{TP_{i}+TN_{i}}{TP_{i}+FP_{i}+TN_{i}+FN_{i}} \end{array}$$

where,

True Positive (TP_i_): the number of samples correctly identified as class C_i_;

True Negative (TN_i_): the number of samples correctly identified as any class except the class C_i_;

False Positive(FP_i_); the number of samples incorrectly identified as class C_i_;

False Negative(FN_i_); the number of samples belonging to class C_i_ but incorrectly assigned to other classes;

The overall accuracy, precision and recall was then calculated as their average over three classes.

### Statistical analysis

The mean and standard error of the mean (mean ± SEM) was reported for each variable in each analyzed class. We used a semi-parametric method entitled as generalized estimating equation (GEE) and the multiple comparison *post-hoc* test to identify whether neuron types are associated with repeated responses of action potential shape parameters or their principal components (i.e., extracted parameters of different spikes of each neuron, Hardin and Hilbe, 2003). The Q-Cochran's test and the McNemar's *post-hoc* test were used to identify whether one classifier statistically significantly outperforms the others (Webb and Copsey, 2011). The Bonferroni correction was used for multiple comparisons and the adjusted *P-*values were then used for interpretation. The level of significance was set to 0.05.

The feature extraction, clustering and classification were performed using MATLAB R2017b (Mathworks, Natick, MA). The statistical analysis was performed using the IBM SPSS Statistics for Windows, version 22.0 (Armonk, NY: IBM Corp.). The developed programs, and the recorded datasets are entirely available online at https://doi.org/10.6084/m9.figshare.6739514.

## Results

A number of 14 (5 SST, 4 PV, and 8 Pyr) neurons were used in our experiment. We extracted 788, 849, and 2,989 spikes from pyramidal, somatostatin and parvalbumin neurons, respectively. Figures 2C,F,I shows the DCT coefficients for three types of neurons. In this Figure, The DCT coefficients of various type of neuron are rather different. The value of the J_3_ index as a measure of class separability for DCT features and raw signals were 191.5 and 72.09, respectively showing better separability of the DCT features.

**Figure 4:**
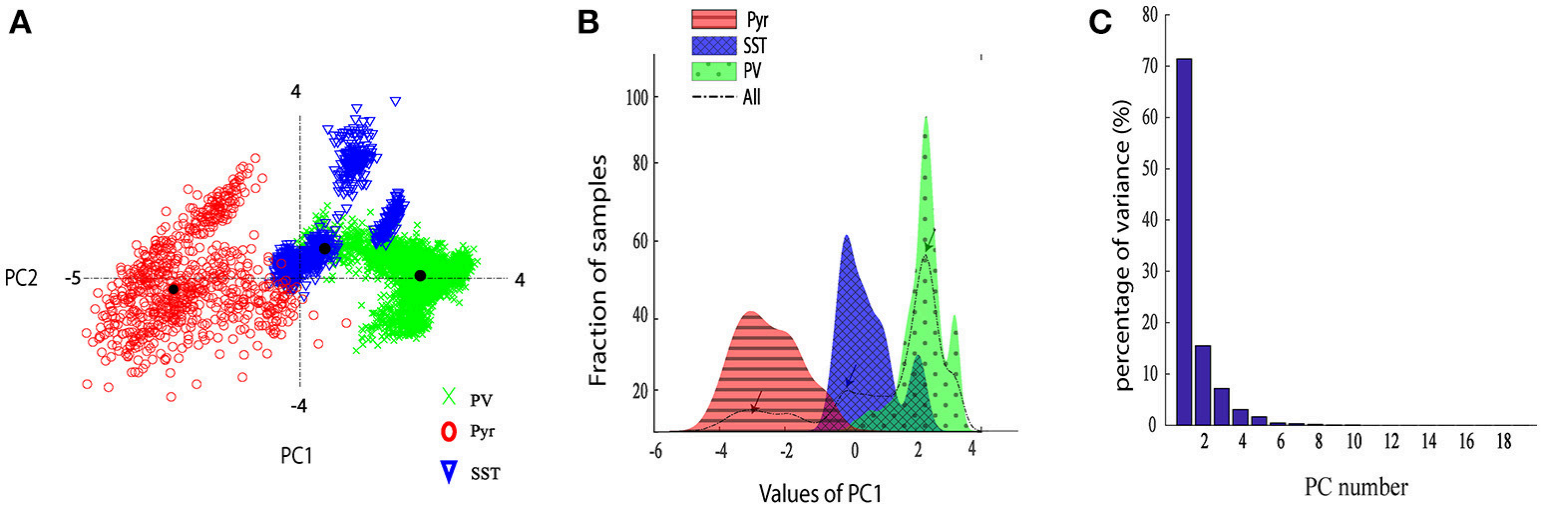
Neurons discrimination based on PCA. **(A)** The scatter plot of the two PCs of the DCT of the spikes. Different groups are marked with different colors and marker. **(B)** The histogram of the first PC of the DCT coefficients. Overall the dashed black line shows sample distribution and different groups are marked with different colors; **(C)** The Percentage of variance captured by each PC, PCs 1–20.

In order to explore the variability of DCT coefficients extracted from the neuron spikes, we provided the scatter plot of their first two principal components (Figure 4A). Moreover, Figure 4B shows the distribution of the first PC space of different classes. The dashed black line displays the total distribution of samples while the distribution for each type of neuron is plotted by a corresponding color. It is apparent that the total distribution (dash line) is clearly Tri-modal. The first peak corresponds well to the Pyr neuron type, while the second and third ones correspond SST and PV neurons, respectively.

**Figure 5:**
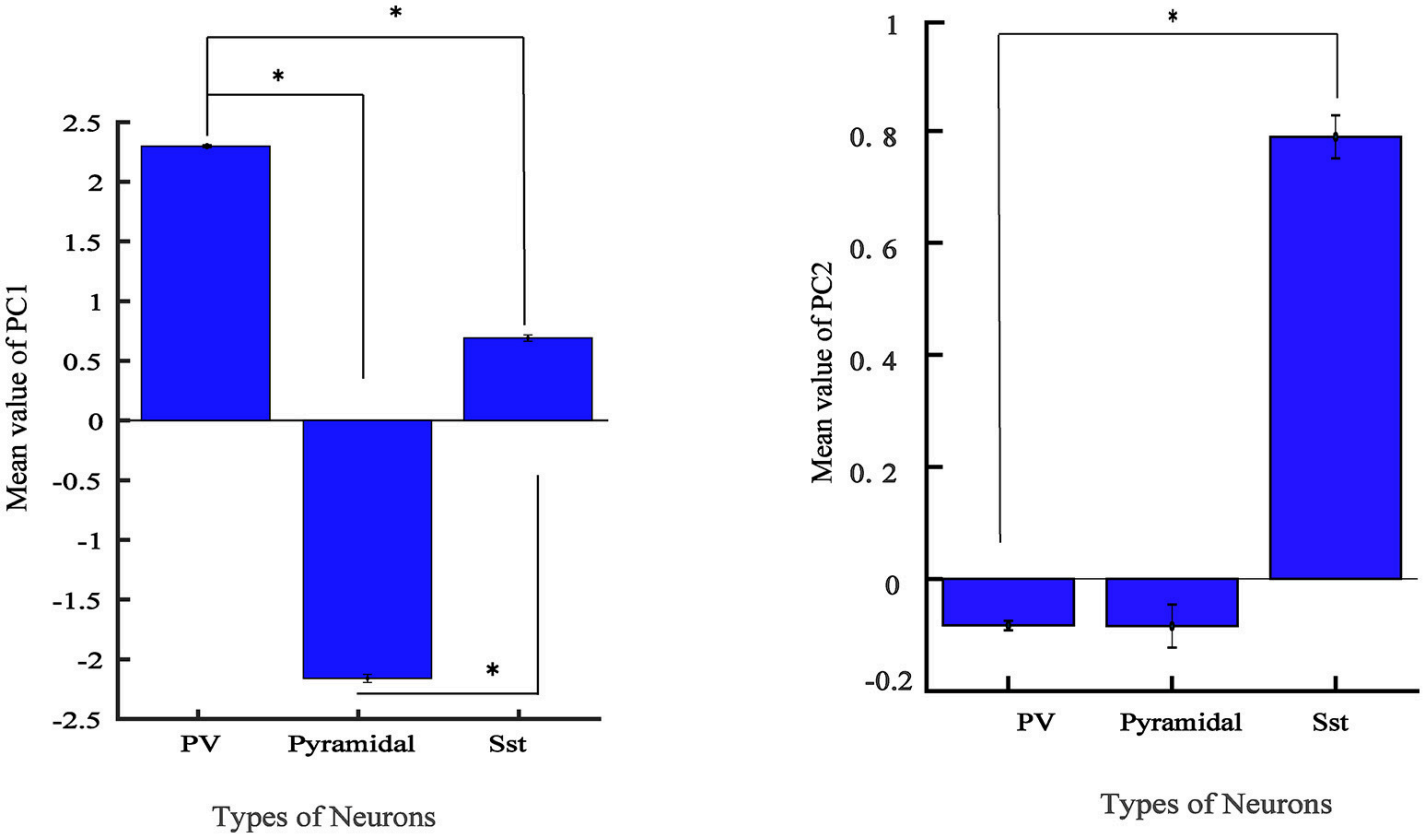
Values of feature in the first 2 PCs in three electrophysiological classes of neurons in mice V1. Bar graph shows the mean values of PC for different groups and error bar shows the SEM value. (^*^adjusted *p*_value_ < 0.05, GEE).

From Figure 4A, it is apparent that two visible clusters can be accounted for the SST interneuron while no clear sub clusters were observed in PCA space for the other two clusters. In fact, the second peak in the distribution of SST neurons (Figure 4B; color blue) has a significant overlap with the PV distribution function. This may occur for two reasons: first, the projection chosen to plot by the first two PCs causes these types to appear mixed while, being separated in the entire high-dimensional feature space. Second, maybe they belong to different subtypes of SST interneurons as investigated in the previous study (McGarry et al., 2010). The PV group encompassed approximately half of the neurons and was highly separated from the Pyr neurons cluster (red and green; Figure 4A). Nearly all spikes of Pyr neurons (red distribution) had negative values in the first PC, whereas SST had slightly positive and near-zero values and PV cells (green) had more positive values. The percentage of the variability represented by each PC is shown in Figure 4C. In our study, the first two PCs were selected (73.78% for PC1 and 15.52% with PC2: total 89.3%). We further statistically explored the variability of the retained PCs. The PCs of the three groups were significantly different (Figure 5). In fact, all groups had significant differences with each other using either PC1 whereas in PC2 just PV and SST has significant difference (*p* < 0.05).

**Figure 6:**
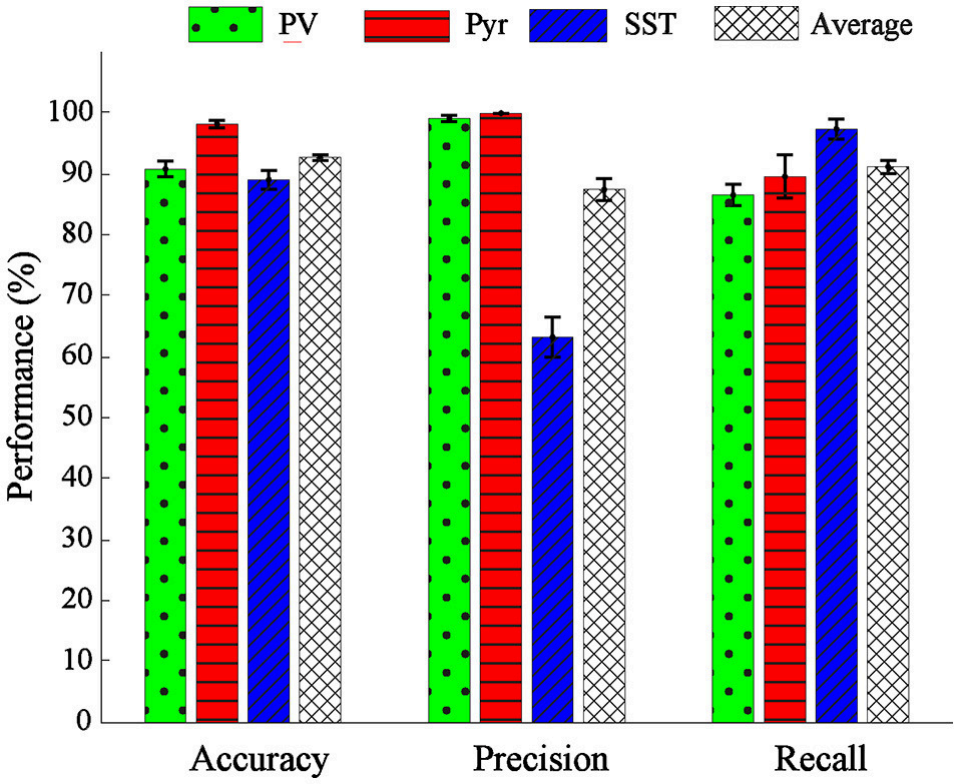
Performance evaluation in terms of accuracy, precision, and recall. The performance of classification was shown for single classes of PV (green bar), Pyr (red bar), and SST (blue bar) and also their overall mean (black dashed bar).

The overall performance of the proposed classification method was reported in mean ± SEM over 10-folds (Figure 6). The overall accuracy, precision, and recall were 92.67 ± 0.54, 87.13 ± 2.59, 87.05 ± 0.74%, respectively (rightest bars in each group in Figure 6) confirming the relative discriminant power of the extracted features. We also calculated the performance of the classification in each class. The accuracy, precision and recall values were 91.59 ± 1.18, 95.45 ± 0.54, 91.33 ± 1.91, for PV; 97.42 ± 0.69, 100 ± 0.00, 84.89 ± 4.11% for Pyr and 89.01 ± 1.12, 65.68 ± 3.15, 84.69 ± 1.98% for SST classes. The classification accuracy of Pyr group is higher than that of the other two groups. This is in fact in agreement with the visual interpretation of the distribution and the scatter plot of the PCs in Figure 4.

**Figure 7:**
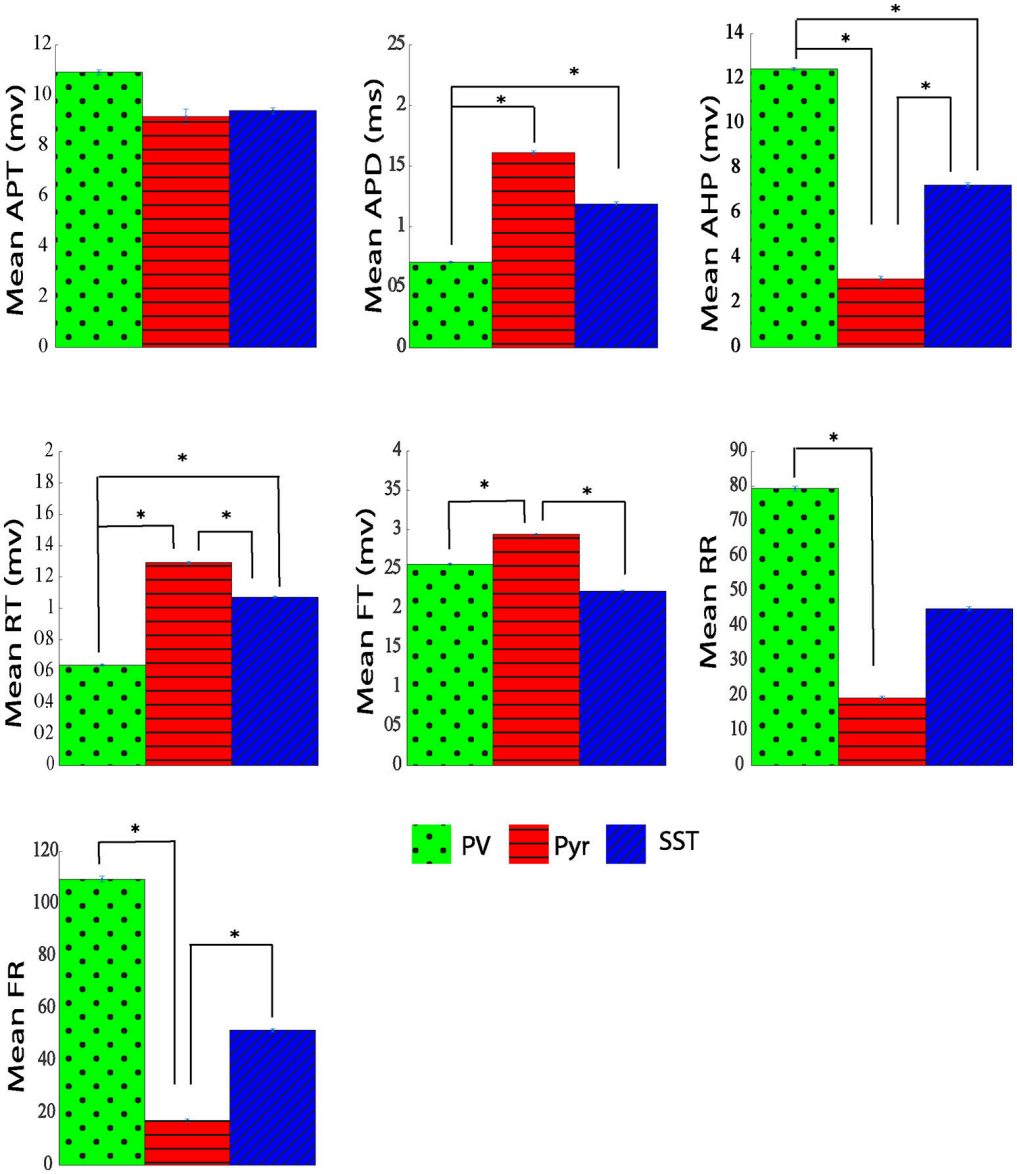
The Comparison of physiological parameters extracted from action potential shape for three different types of neurons. All data shown mean ± standard error (APT, action potential threshold; APD, action potential duration; AHP, After hyperpolarization; RT, rise time; FT, fall time; RR, rise rate; FR, fall rate. ^*^adjusted *p*_value_ ≤ 0.05, GEE).

The average and dispersion of the extracted parameters from action potential shape were shown in each neuron group (Supplementary Table S1 and Figure 7). The performance of that method was assessed using the 10-fold cross-validation with the same folds as our analysis. Extracting all 7 parameters, we performed PCA and retained the first two PCs (CPV of 90%). The overall accuracy was 82.29 ± 1.31%. The accuracy for PV, Pyr, and SST identification was 75.20 ± 4.24, 91.40 ± 1.92, and 80.27 ± 4.16%, respectively. Our proposed neuron classification system significantly outperformed this classification system (McNemar's test; *p* < 0.05).

Moreover, in order to assess the performance of our method on other experiments such as *in vitro* whole cell recording, the program was further tested on the pool of 50 neurons in the Allen Cell Types Database (Allen Cell Types Database, 2015). We used the data from 5 major subtypes of neurons namely as excitatory pyramidal neuron, and four major inhibitory neurons namely as parvalbumin, somatostatin, 5HT3a and the vasoactive intestinal polypeptide (VIP) expressing neurons. Transgenic mice lines Nr5a1-Cre, Pvalb-IRES-Cre, Sst-IRES-Cre, Htr3a-Cre_NO154, and Vip-IRES-Cre were selected to target pyramidal, PV, SST, 5HT3a, and VIP neurons, respectively. We used 10 neurons per each group from layers 2/3,4,5,6a of the cortex. The number of extracted spikes for Pyramidal, PV, SST, 5HT3a, and VIP groups were 334, 721, 447, 378, and 459, respectively. Using five-fold cross validation, with one principal component after dimension reduction, the neuronal classes were predicted with the overall accuracy of 84.13 ± 0.81%. The PV class was the best predicted class (93.57 ± 0.59%), followed by the SST (89.15 ± 0.63%), VIP (81.69 ± 0.56%), 5HT3a (79.23 ± 1.38%), and Pyr (77.02 ± 0.91%). The exported *in vitro* dataset and the developed program could be assessed in the file “*in_vitro*.zip” located at https://doi.org/10.6084/m9.figshare.6739514.

## Discussion

Our main finding in this study is the efficacy of *in vivo* whole-cell intracellular recording in the electrophysiological classification of neural subtypes. This approach would be useful for laboratories with intracellular recordings *in vivo*, especially for recording from deep structures, where visualization is challenging and alternative approaches such as blind intracellular recording is an appropriate option. There are some alternative options to understand cell-type such as optogenetics tagging by Optopatcher or Optrode by using optical fibers (Kravitz et al., 2013; Muñoz et al., 2017). In the literature, most of the classification methods have been performed on the base of neural spiking data and extracellular recording technique but because of limitations in identifying neural action potential characteristics, the efficacy of these methods is limited (Lima et al., 2009; Moore and Wehr, 2014). In single unit recordings, a limited number of features such as spike half-width, energy of signal and so on are widely used for neuron classification (Quirk et al., 2009; Li et al., 2015). To the best of our knowledge, there were not any systematic approaches similar to our study in the literature. Although most of the researchers believe that it is not enough to rely on electrophysiological features of the neurons for their classification, we showed that it is possible to use intracellular recordings for such a purpose. However, the identification accuracy of the proposed method requires improvement.

This approach would be particularly beneficial in blind intracellular recordings in animal models and in operation rooms for a better understanding of neural functions in the brain, e.g., in epileptic patients that surgical operations and corticectomy are treatment choices (Jobst, 2012; Rey et al., 2015). Moreover, classifying cell types was subjective leading to inconsistent identification of neurons before. Lately, clustering methods were used for this identification (Cauli et al., 1997; Karube et al., 2004; Ma et al., 2006; Dumitriu et al., 2007; Helmstaedter et al., 2009; Karagiannis et al., 2009; DeFelipe et al., 2013).

We not only used *in vivo* data to test the proposed algorithm, but we also analyzed *in vitro* data to identify whether it works under other experimental conditions. In fact, different cortical layers and number of classes were distinctive factors in our analysis. The overall accuracy of our program was 92.67 ± 0.54 and 84.13 ± 0.81% using cross validation framework for *in vivo* and *in vitro* datasets, respectively. While in the former case, we had three classes, five classes were analyzed in the latter case. The Pyr was the best predicted class (97.42 ± 0.69%) in the *in vivo* study while the PV had the highest identification accuracy (93.58 ± 0.91%) in *in vitro* dataset. We recorded the *in vivo* data while the Allen Cell Types Database was used for *in vitro* analysis. The proposed program is robust, since it works well on both *in vivo* and *in vitro* datasets without any systematic modifications.

Several studies have been performed on the classification of different neuronal subtypes using Allen Cell Types Database. For example, Gouwens and colleagues used a supervised method to classify neurons into putative types corresponding to four neuronal types (Pyr, PV, SST, and 5HT3a; Gouwens et al., 2018). The overall accuracy of the classification using 12 electrophysiological features and support vector machine classifier was 79%. The Pyr class was predicted with the accuracy of 91% followed by the PV (80%), SST (59%), and 5HT3a (50%) classes. In comparison, we identified PV, SST, VIP, 5HTa, and Pyr with the accuracy of 93.57 ± 0.59, 89.15 ± 0.63, 81.69 ± 56, 79.23 ± 1.38, and 77.02 ± 0.91%, respectively. We outperformed the classification method proposed by Gouwens and colleagues in identifying PV, SST, and 5HT3a classes but not Pyr.

Various supervised and unsupervised methods can be used to examine neuronal feature subtypes (Li et al., 2015). In fact, a promising way to understand cortical circuit functions is the classification of neural types based on different properties. In the past, criteria were often qualitative and were not standardized. Therefore, more challenges remain in the classification of interneurons and finding a set of relevant descriptors. Several groups have used clustering algorithms for identifying various interneurons subtypes (Cauli et al., 2000; Karube et al., 2004; Ma et al., 2006; Dumitriu et al., 2007; Helmstaedter et al., 2009). Frey and Dueck (2007) devised the affinity propagation, that significantly improved results over standard methods. It was also shown in another study that the k-means algorithm had better performance than Ward's method to classify four subtypes of PV interneuron (Helm et al., 2013). They used the average within-cluster distance, the average between-cluster distance, and the Calinski-Harabesz index to compare Ward's and k-means cluster results. Such unsupervised methods, already resulting in high identification accuracies, could be improved if combined with dimensionality reduction (Guerra et al., 2011). Gentet et al. (2010) investigated the membrane potential dynamics of excitatory and inhibitory neurons in the barrel cortex of behaving mice. They used 2-photon targeted whole-cell patch-clamp recordings to measure parameters from spike waveform of neuron such as action potential duration, input resistance, mean membrane potential, and action potential frequency. The neurons were divided into three main groups, namely as excitatory, fast spiking, and non-fast spiking inhibitory neurons. Based on their results, excitatory neurons (i.e., pyramidal neuron), fast spiking (e.g., PV), and non-fast spiking (e.g., SST) inhibitory neurons have longest to shortest action potential duration. This result is in agreement with our measurement from action potential duration of neurons. Jouhanneau et al. (Jouhanneau et al., 2018) represented similar results for Pyr, SST, and PV neurons. Also, they measured the action potential threshold as a distinctive parameter in different cell types. Being in good agreement with our measurement, their results showed that the PV neuron has the highest and the SST group has lowest value of action potential threshold.

Our study showed that the mean rise time was significantly shorter in PV neurons compared with both Pyr and SST neurons. However, there is not a significant difference between Pyr and SST neurons rise time. The rising rate for PV neurons was significantly larger (near 50%) compared with SST, and Pyr neurons, suggesting a higher sodium channel density for PV cells. On the other hand, the falling rate of PV neurons is also larger (at least by 50%) compared with that of the other two groups. It may suggest a higher potassium channel density for PV neurons. However it has been shown in literature that difference in spike duration is dependent on passive properties of neurons such as time constant (in PV+ neurons are less than SOM+ neurons; Stuart et al., 1997; Kowalski et al., 2016; Safari et al., 2017) as well as active properties of neurons such as amount of Kv3 potassium channels (Bean, 2007). PV neurons have a larger after hyperpolarization amplitude compared with Pyr and SST groups (12.45 ± 0.07 mV vs. 3.06 ± 0.11 and 7.23 ± 0.1 mV). Comparing the action potential duration (APD) of the three groups of neurons revealed three distinct groups. Both Pyr and SST cells have longer-duration action potentials with 1.41 ± 0.012 ms and 1.18 ± 0.01 ms, respectively, in comparison with PV neurons which has shorter action potential duration (0.70 ± 0.005 ms).

In the current study, we used a limited number of neurons. Increasing the sample size will improve the statistical power of our classification. We did not add some *in vivo* functional features such as inter-spike interval (ISI) or firing pattern of neurons in our method. Adding such features would be beneficial as to increase the performance of the classification. Moreover, further morphological features could be extracted to improve the neuron classification. They will be the focus of our future works. Our method still is not able to predict all of the correct samples (with the overall accuracy of 92.67 ± 0.54 and 84.13 ± 0.81% on *in vivo* and *in vitro* data, respectively) but the identification rate is higher than that of the-state-of-the-art.

## Conclusion

We proposed a semi-supervised method to identify three subtypes of neurons in layers 1 and 2/3 of the visual cortex of transgenic mice using *in vivo* whole-cell patch-clamp data. The DCT transform and previously proposed electrophysiological parameters were used to classify three subtypes of cortical neurons. We showed that the DCT features are more informative than extracted electrophysiological parameters. In addition to the application of our method to identify *in vivo* neuronal subtypes, its application to classify 5 major neuronal subtypes for *in vitro* data was presented. We used a considerable pool of *in vitro* data available online from Allen Database. Although the performance of the proposed method was lower in *in vitro* data compared with *in vivo*, it outperformed the stat-of-the-art in this field. The proposed method is thus promising in identifying cell types based on the electrophysiological characteristic of the neurons.

## Author contributions

M-SS designed the experiments, performed the *in vivo* recordings, and participated in conceptualization. PG and HM participated in formal analysis, investigation, and methodology. PG and M-SS, contributed to writing the original draft while HM revised the manuscript. M-SS participated in project administration and resources and acquired funding. All authors read and approved the final version of the manuscript and agreed for all aspects of the work.

### Conflict of interest statement

The authors declare that the research was conducted in the absence of any commercial or financial relationships that could be construed as a potential conflict of interest.
