# Supplementary material for: Electrophysiological Profiling of Neocortical Neural Subtypes: A Semi-Supervised Method Applied to in vivo Whole-Cell Patch-Clamp Data
Source: Front Neurosci. 2018 Nov 13;12:823. doi: 10.3389/fnins.2018.00823 (PMC6277855; doi:10.3389/fnins.2018.00823)
Supplement: Supplementary file 1 [file Table_1.DOCX]

Supplementary Material

Electrophysiological Profiling of Neocortical Neural Subtypes: A Semi-Supervised Method Applied to In vivo Whole-cell Patch-clamp Data

Parviz Ghaderi^1^, Hamid Reza Marateb^2*^, Mir Shahram Safari^1,3*^

*** Correspondence:**

Dr. Mir Shahram Safari
[safari@sbmu.ac.ir](mailto:safari@sbmu.ac.ir)

Dr. Hamid Reza Marateb
[h.marateb@eng.ui.ac.ir](mailto:h.marateb@eng.ui.ac.ir)

**Table S1.** **The comparison of different extracted parameters from the action potential shape in different neuron classes. Values are reported as mean ± standard error of mean (SEM).**

| Variable | PV group | Pyr group | SST group |
| --- | --- | --- | --- |
| Normalized AP threshold (mV) | 10.91±0.100 | 9.15± 0.291 | 9.39±0.110 |
| AP duration (ms) | 0.70±0.005 | 1.61±0.016 | 1.18±0.010 |
| AP Hyperpolarization (mV) | 12.45±0.070 | 3.06±0.118 | 7.23±0.100 |
| AP rise time (ms) | 0.63±0.005 | 1.29±0.007 | 1.07±0.003 |
| AP fall time (ms) | 2.55±0.008 | 2.93±0.004 | 2.21±0.010 |
| AP rise rate(mV/ms) | 89.34±0.770 | 19.29±0.608 | 44.91±0.620 |
| AP fall rate(mV/ms) | 109.42±1.150 | 17.13±0.558 | 51.66±0.620 |
